# An adapted algorithm for patient engagement in care for young people living with perinatal HIV in England

**DOI:** 10.1186/s12913-023-10122-5

**Published:** 2023-10-18

**Authors:** Marthe Le Prevost, Deborah Ford, Siobhan Crichton, Caroline Foster, Alasdair Bamford, Ali Judd, Adolescents and Adults Living with Perinatal HIV (AALPHI) Steering Committee

**Affiliations:** 1 MRC Clinical Trials Unit at University College London, 90 High Holborn, 2nd Floor, London, WC1V 6LJ UK; 2 Imperial College Healthcare NHS Trust, London, UK; 3 Great Ormond Street Hospital for Children NHS Foundation Trust, London, UK

**Keywords:** Engagement in care, Perinatal, HIV, Young people, Adolescents, England

## Abstract

**Background:**

Evidence suggests that engagement in care (EIC) may be worse in young people living with perinatal HIV (YPLPHIV) compared to adults or children living with HIV. We took a published EIC algorithm for adults with HIV, which takes patients’ clinical scenarios into account, and adapted it for use in YPLPHIV in England, to measure their EIC.

**Methods:**

The adult algorithm predicts when in the next 6 months the next clinic visit should be scheduled, based on routinely collected clinical indicators at the current visit. We updated the algorithm based on the latest adult guidelines at the time, and modified it for young people in paediatric care using the latest European paediatric guidelines. Paediatric/adolescent HIV consultants from the UK reviewed and adapted the resulting flowcharts. The adapted algorithm was applied to the Adolescent and Adults Living with Perinatal HIV (AALPHI) cohort in England. Data for 12 months following entry into AALPHI were used to predicted visits which were then compared to appointment attendances, to measure whether young people were in care in each month. Proxy markers (e.g. dates of CD4 counts, viral loads (VL)) were used to indicate appointment attendance.

**Results:**

Three hundred sixteen patients were in AALPHI, of whom 41% were male, 82% of black African ethnicity and 58% born abroad. At baseline (time of AALPHI interview) median [IQR] age was 17 [15–18] years, median CD4 was 597 [427, 791] cells/µL and 69% had VL ≤50c/mL. 10 patients were dropped due to missing data. 306 YPLPHIV contributed 3,585 person months of follow up across the 12 month study in which a clinic visit was recorded for 1,204 months (38/1204 dropped due to missing data). The remaining 1,166 months were classified into 3 groups: Group-A: on ART, VL ≤ 50c/mL—63%(734/1,166) visit months, Group-B: on ART, VL > 50c/mL—27%(320/1,166) Group-C: not on ART-10%(112/1,166). Most patients were engaged in care with 87% (3,126/3,585) of months fulfilling the definition of engaged in care.

**Conclusions:**

The adapted algorithm allowed the varying clinical scenarios of YPLPHIV to be taken into account when measuring EIC. However availability of good quality surveillance data is crucial to ensure that EIC can be measured well.

**Supplementary Information:**

The online version contains supplementary material available at 10.1186/s12913-023-10122-5.

## Introduction

High engagement in care (EIC) is recognised as a crucial step in improving the outcomes of people living with human immunodeficiency virus (HIV) [1–4]. Adult studies have found a higher likelihood of HIV viral suppression and improved CD4 cell counts in people who have better EIC [5, 6] and higher EIC has been associated with lower healthcare costs [4, 7, 8]. Furthermore, transmission of HIV may be more frequent in people who are not engaged in care [9, 10].

A number of studies have found that young people (YP) living with HIV have worse EIC compared to adults and children [5, 9, 10]. In a national study of 72,218 adults living with HIV in the UK, those aged 15–24 years were the most likely to be lost to follow-up [11]. In another study of 87,146 people living with HIV across all age groups in New York, a U-shaped relationship between age and EIC was found, with people at the youngest (birth to 12 years) and oldest (60 years and older) age ranges having the best EIC, and YP aged 20–29 years the lowest, across the whole age spectrum [9].

However, there is considerable variability in how these measures are defined, in terms of the types of visits considered as either missed or attended (e.g. appointments with the doctor, nurse, psychologist or phlebotomist), and visit frequency (e.g. one visit in 6 months or one visit in a year). There is also a wide variation in the health status of people living with HIV, and those with worse clinical indicators are likely to be scheduled to be seen more frequently. For these patients, use of a simple EIC measure such as a visit frequency of at least one visit in a 6 month period might be obtained but be misleading, and fail to capture missed visits within the period.

Additionally, measuring EIC across the transition period is further complicated because of differences in data sources and guidelines. YP living with perinatal HIV (YPLPHIV) may receive HIV care in paediatric as well as adult HIV services. This requires linkage of paediatric and adult datasets, which may be held separately and by different data controllers. Additionally, whilst many YP seen in paediatric clinics have clinic visits every three to four months [12], older patients who are stable on antiretroviral therapy (ART) may have clinic visits every 6 months, in accordance with adult HIV guidelines [13]. Thus application of a simple definition of EIC to YPLPHIV, with the same length of gap applied between visits, would not account for these nuances and changes over time including transition to adult care.

In this paper, our aim was to account for the complexity of EIC in YPLPHIV by developing a new measure. We describe how we took a published EIC algorithm for adults with HIV, originally developed by Howarth et al. [14], and adapted it for use in YPLPHIV in England. Howarth et al.*’s* rationale for developing this new algorithm was that standard EIC measures do not take into account how the frequency of clinic attendance is based on individual patients’ health and treatment status and that this changes over time [14]. Their algorithm used clinical factors to predict when adult patients should next be seen in clinic (i.e. the predicted next appointment date). We updated Howarth et al.*’s* definitions to more recent adult treatment guidelines and modified appointment scheduling for young people still in paediatric care based on the latest European paediatric guidelines [12, 13]. This adapted algorithm was then applied to a dataset of YPLPHIV in England in the Adolescents and Adults Living with Perinatal HIV (AALPHI) cohort [14]. We hope that findings from this work can be used to help create a risk assessment that can be used to identify those at higher risk of disengaging from care for additional support.

## Methods

The AALPHI cohort study was a prospective study in which a wide range of data were collected to describe the impact of life-long HIV and long-term ART on health outcomes. YPLPHIV (aged 13–21 years) were recruited from NHS clinics and voluntary sector organisations across England, and were interviewed at two different time points between 2013 and 2017. Detailed AALPHI methods have been described previously [15, 16]. Ethical approval was obtained from Leicester Research Ethics Committee. All AALPHI participants living with PHIV were also in the national UK Collaborative HIV Paediatric Study (CHIPS) [17], which captures the clinical data used in this analysis.

The Howarth algorithm was updated to the most recent British HIV Association (BHIVA) clinical monitoring guidelines at the time, [13] and then reviewed alongside the Penta paediatric HIV treatment guidelines [12]. Where Penta guidelines recommended a shorter maximum time to next appointment for a group of patients, the algorithm was updated accordingly [17]. The adapted algorithm was then reviewed with two clinicians caring for YPLPHIV in large London clinics, as well as the Steering Committee of CHIPS. Three main groups of YPLPHIV were identified. Within each group, a decision tree approach was taken to predict the next scheduled appointment date, with the following variables used to predict the next appointment: viral load (VL), CD4 cell count, CDC C (AIDS defining) events, weight, ART start/new regimen, number of ART drugs, time on ART, third agent (protease inhibitor (PI)/non-nucleoside reverse transcriptase inhibitors (NNRTI)), care provision (paediatrics/adult).

### Application of the YPLPHIV algorithm to a clinical dataset

AALPHI first interviews were conducted between 2013 and 2015. The date of the first AALPHI interview was treated as the start date for this analysis and follow-up time was for one year. Data were available from CHIPS for the duration of follow-up and from the clinic visit before the AALPHI interview date for participants who did not have an interview and clinic visit on the same date (to predict the date of their first visit in the follow-up period). As actual clinic visit dates were not collected in CHIPS, dates of clinical markers routinely collected in clinic appointments were used as proxy clinic visit dates, following the approach used by others [14, 18]. Clinical markers used were CD4 cell count, VL, weight, and height, ART start and ART switch (defined as on continuous therapy but a component drug changed).

For each attended visit (proxy clinic visit) EIC flowcharts were used to predict time to next visit (1, 2, 3, 4, or 6 months). Based on this prediction, each month following was defined as in or out of care, until the next month including an attended visit. For any month which included a predicted visit, a participant was considered in care if they attended clinic early and prior to the start of the month, or if they attended clinic within the month. Where there were multiple visits in the same month, information from the latest visit was used to predict the time of the next visit. Where proxy visit dates were available but clinical data (VL and CD4 cell counts) were not complete enough to estimate the time to next predicted visit, missing data were imputed. In these instances, the last measurement (VL or CD4) was carried forward for up to six months to correspond to the longest time between appointments in the flowcharts. For clinic visits where data were not available in the previous 6 months, the clinical data were reviewed and, where clinically appropriate, imputation criteria were defined to carry data forward for longer than 6 months. Patients who had no CHIPS data for the whole follow-up year could not be classified in the flowcharts and were therefore excluded.

**Fig. 1:**
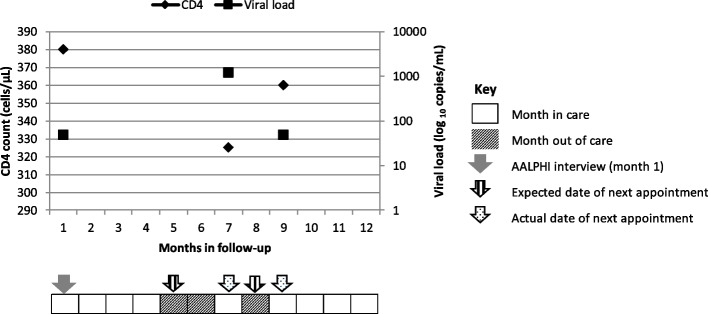
Months in and out of care for a hypothetical participant in paediatric care adapted from Howarth et al. [14].

Figure 1 shows months in and out of care for a hypothetical participant in paediatric care. This participant has a clinic visit on the day of their AALPHI interview (with VL ≤ 50c/mL and CD4 = 380 cells(c)/µL) (month 1). They are classified using the Group A Flowchart with a predicted visit in month 5. They attend in month 7, so are considered in care for four months and out of care for two months. At the clinic visit in month 7 (VL = 1,200c/mL and CD4 = 325c/µL), they are classified using the Group B Flowchart and their predicted visit is in 1 months’ time. They attend after two months so are considered in care for month 7 and out of care for month 8. When they attend in month 9 (VL ≤ 50c/mL and CD4 = 360c/µL), they are again classified using the Group A Flowchart, with the predicted visit in 4 months’, so they are considered in care for the remaining time. Overall, the participant has nine months classified as engaged in care and three months classified as out of care.

We first describe the months in which a visit took place (referred to as "visit months"), and summarise the frequencies of predicted follow-up intervals to next appointment (1 to 6 months). We describe the distribution of predicted follow-up intervals by participant and estimate the median number of attended visits per participant. We then compare actual visit intervals to predicted visit intervals; for this analysis, we dropped the last predicted appointment for all participants (to avoid addressing scheduled visits post end of follow-up); we considered visits attended one month early as "on time" (to allow for scheduling within busy NHS clinics), thus we looked at visit intervals of ≥ 3 months. Finally, total time ‘engaged in care’ was calculated as the total number of months classified as in care. All data preparation, cleaning and analysis were carried out in STATA version 15 [19].

## Results

Of 316 patients in AALPHI, 41% (*n* = 129) were male, 82% (*n* = 258) of black African ethnicity and 58% (*n* = 184) were born outside the UK/ Ireland (Table 1). Twenty seven per cent of participants (*n* = 84) had a previous CDC C event. At baseline (time of AALPHI interview), median [IQR] age was 17 [15-18] years, median CD4 count was 597 [427, 791] and 69% (*n* = 202) had viral load ≤ 50c/ml.

**Table 1:** Characteristics of AALPHI participants at time of interview

Variable and categories	Number (%) or median [interquartile range (IQR)] (*n* = 316)	
Male sex	129	(41%)
Age (years)	17	[15, 18]
Ethnicity:		
Black African	258	(82%)
Black other	13	(4%)
Mixed	30	(10%)
White	9	(3%)
Asian	4	(1%)
Born outside UK/Ireland	184	(58%)
HIV severity:		
Previous CDC C event	84	(27%)
Nadir CD4 cell count (cells/µL)	221	[121, 352]
CD4 cell count (cells/µL)^a^	597	[427, 791]
Viral load ≤ 50c/mL^b^	202	(69%)
ART:		
On ART	279	(88%)
Age of ART start	7	[3, 12]

^a^Data only available on 279 participants at the time of the AALPHI interview

^b^Data only available on 291 participants at the time of the AALPHI interview

Where a clinic visit did not take place on the interview date, clinical data from the most recent visit prior to the AALPHI interview were used

Across the 12 months following the AALPHI interview, 10 participants were excluded due to having no clinical (CHIPS) data. For the remaining 306 participants, there were 3,585 months of follow-up, in which a clinic visit was recorded in 34% (*n* = 1,204) months. Following imputation, 3% (38/1,204) visit months remained unclassified due to incomplete ART and/or clinical information. These 38 were coded as in care for one month (the minimum time to next scheduled appointment). If the participant did not attend within this month, then subsequent months without a visit were dropped until the participant attended again. The remaining 1,166 visit months were classified into three main groups as follows:

- Group A: on ART with viral load ≤ 50c/mL: 63% (734/1,166)
- Group B: on ART with viral load > 50c/mL: 27% (320/1,166)
- Group C: not on ART (ART naïve or stopped ART): 10% (112/1,166)

The flowcharts for groups A-C show the clinical decisions used to predict a participant’s next appointment with timings of the next scheduled appointment indicated in all the shaded terminal nodes.

### Group A Flowchart: YPLPHIV on ART with viral load ≤ 50c/mL

**Fig. 2:**
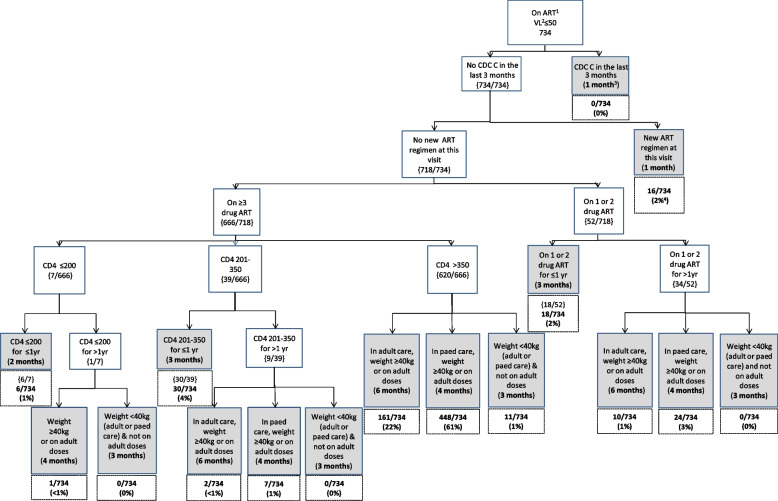
Group A Flowchart—visits in YPLPHIV on ART with viral load ≤ 50c/mL (participants = 235). Footnote: ^1^ART = Antiretroviral therapy, ^2^VL = viral load, ^3^Months to next scheduled appointment, ^4^Proportions given at the terminal nodes of decision trees. As an example, if a participant had no CDC C events in the preceding three months, has no change to ART drugs at this visit, is on ≥ 3 drug ART regimen, and has a CD4 of between 201-350c/µL for less than a year, their next predicted appointment would be in three months’ time

Figure 2 shows that 235 participants attended one or more visits on ART with a viral load ≤ 50c/mL, contributing a total of 734 visit months. There were no CDC C events in the last 3 months, and no change in ART prescribed in 98% (*n* = 718) of visit months. In 2% (*n* = 16) of visit months, the participant had started a new ART regimen, meaning their next predicted clinic visit was 1 month later. Of the remaining 718 visit months, 7% (*n* = 52) were on 1- or 2-drug ART, and the majority of visit months, 93% (*n* = 666), were in participants who had not started new ART and were on 3 drug ART. Overall, at 65% (480/734) of visits in Group A, the predicted time to next appointment (based on clinical characteristics) was in 4 months’ time and at 24% (*n* = 173) it was in 6 months’ time.

### Group B Flowchart: YPLPHIV on ART with viral load > 50c/mL

**Fig. 3:**
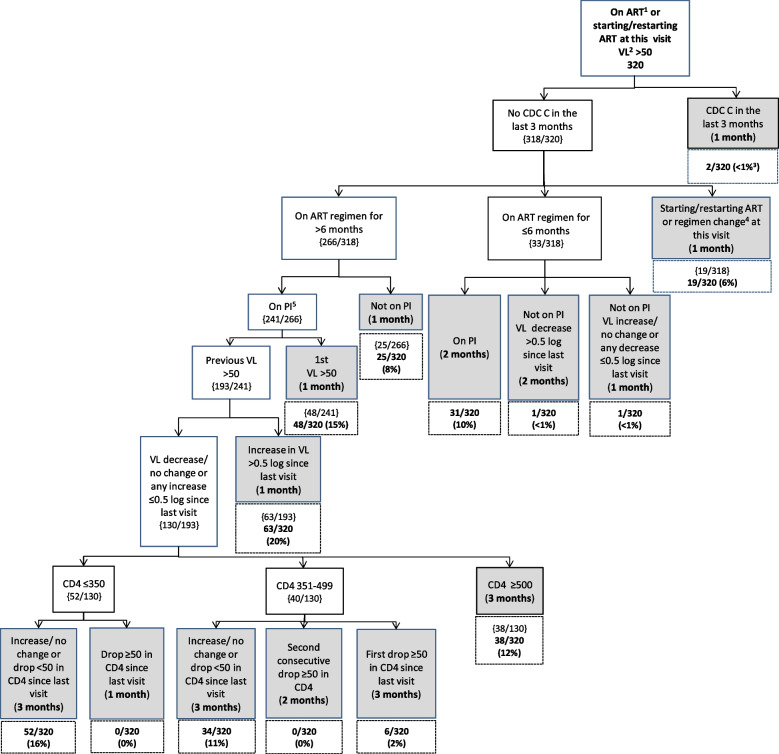
Group B Flowchart—visits in YPLPHIV on ART with viral load > 50c/mL (participants = 112). Footnote: ^1^ART = Antiretroviral therapy, ^2^ VL = viral load, ^3^Proportions given at the terminal nodes of decision trees, ^4^Regimen change = on continuous therapy but a component changed, ^5^PI-protease inhibitor

A total of 320 visit months were in 112 participants who were on ART with a detectable viral load (> 50c/mL) (Fig. 3). There were two consecutive visits where the same participant had a CDC C event in the previous three months. At each visit, the model predicted monthly follow-up visits for the participant. Of the remaining 318 visit months, 84% (*n* = 266) were visits where the participant had been on ART for > 6 months, of which 91% (*n* = 241) were visit months where the participant was on a PI based regimen (e.g. boosted darunavir, atazanavir or lopinavir). In 80% (*n* = 193/241) of these visit months, participants had a previous viral load > 50c/mL, of which 67% (*n* = 130) had a viral load decrease, no change or an increase of ≤ 0.5 log since last visit. Of these 130 visit months, 29% (*n* = 38) were in participants with a CD4 count ≥ 500c/µL, 31% (*n* = 40) a CD4 count of 350-499c/µL and 40% (*n* = 52) had a CD4 count of ≤ 350c/µL. Overall, at 49% (158/320) of visits in Group B, the predicted time to next appointment was in 1 month time, and at 41% (*n* = 130), it was in 3 months’ time.

### Group C Flowchart: YPLPHIV not on ART

**Fig. 4:**
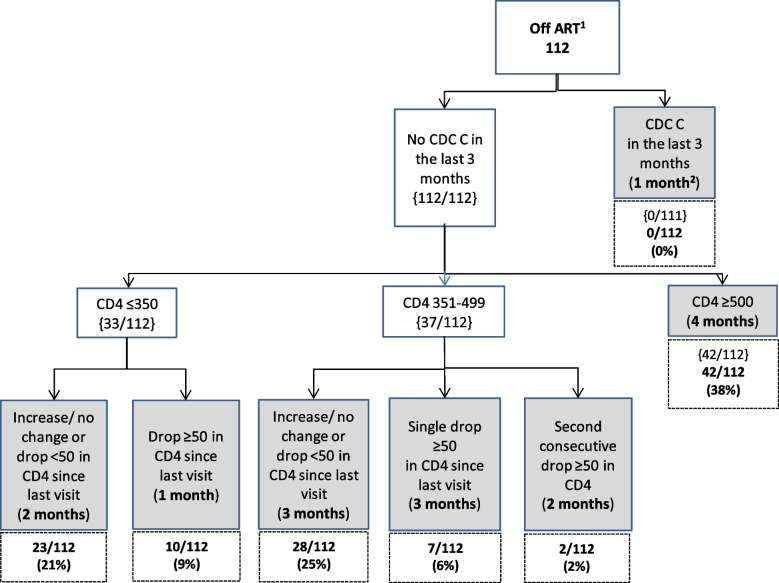
Group C Flowchart: YPLPHIV off ART (participants = 35). Footnote: ^1^ART = Antiretroviral therapy, ^2^Proportions given at the terminal nodes of decision trees

Finally, 112 visits months were in 35 participants who were off ART (Fig. 4). These were a combination of patients who were ART naïve or had had previous ART therapy. None of the visit months were in participants who had a CDC C diagnosis in the last three months. In 29% (*n* = 33) of these off ART visit months, participants had a CD4 count ≤ 350c/µL, in 33% (*n* = 37) of the visit months, participants had a CD4 count 351-499c/µL and in 38% (*n* = 42) visit months participants had a CD4 cell count ≥ 500c/µL. Overall, at 9% (10/112) of visits in Group C, the predicted next appointment was in 1 month’s time, and 38% (*n* = 42) at 4 months’ time.

### Predicted visits and actual appointments

Overall, in 15% (173/1,166) of visit months, the next predicted visit was in 6 months (all in Group A—participants on ART with a VL ≤ 50c/mL). At 45% (*n* = 522) of visit months, the next predicted visit was in 4 months, of which the majority (92%, *n* = 480/522) were in participants in Group A and the remaining 8% (*n* = 42) in Group C (participants who were off ART). At 19% (224/1,166) of visit months, the next predicted visit was in 3 months, of which 26% (59/224) visits were in participants in Group A, 58% (*n* = 130) in participants in Group B (on ART with a VL > 50c/mL) and 16% (*n* = 35) in participants in Group C. Only 5% (63/1,166) of visit months had a predicted visit in 2 months, of which 10% (6/63) were in Group A, 50% (*n* = 32) in Group B and 40% (*n* = 25) in Group C. Finally, in 16% (184/1,166) of visit months, the predicted visit was in 1 month, with 9% (16/184) in Group A, the majority 86% (*n* = 158) in Group B and 5% (*n* = 10) in Group C.

Looking at participants as opposed to visit months, in total, 37% (*n* = 112/306) of participants had at least one appointment during follow-up predicted in 1 month’s time, 9% (*n* = 28) in 2 months’ time, 31% (*n* = 94) in 3 months’ time, with 61% (*n* = 186) in 4 months’ time and 24% (*n* = 73) in 6 months’ time. Overall, 306 participants had 1,166 visits within 12 months of enrollment, equating to a median of 4 [IQR 3, 5] visits per participant per annum.

Participants attended earlier than the predicted appointment in a total of 39% (262/678) of visit months, and on time or late in 61% (*n* = 416). For participants whose next predicted appointment was in 3 or 4 months’ time, around a third of visits were attended early. However, for participants whose time to next predicted appointment was in 6 months’ time, 77% (85/110) of visits were attended early, with most participants attending 2 (23%) or 3 (30%) months early. When the characteristics of participants who attended early for appointments predicted for 6 months were compared to participants who attended on time or late, no major differences were found (data not shown).

Of the 3,585 months of follow-up, 87% (*n* = 3,126) months were classified as in care.

## Discussion

In this paper, we describe how we adapted an existing EIC algorithm for adults living with HIV for YPLPHIV. Penta paediatric and BHIVA adult HIV treatment guidelines were used alongside expert opinion to modify and develop the flowcharts for this population. These adaptations increased the number of different pathways to determine the next predicted appointment from 15 in the adult algorithm to 37 for young people. Although ultimately more complex than the adult algorithm, the YP algorithm is a novel framework which we then applied to a large observational dataset, providing valuable data on EIC.

Engagement in care is commonly measured as a threshold of a specific number of visits per year, and it is generally assumed that a higher number of visits equates to better engagement in care [20–22]. Some studies have simplified this to, for example, one or more visits in a given year, [21, 23] which gives a population estimate useful for public health monitoring and the cascade of care. However, these approaches do not consider the varied and changing clinical scenarios of patients. For a young person who is virally suppressed on ART, two clinic visits per year may indicate an entirely appropriate level of engagement in care. For a young person who does not take their ART and has a low CD4 count, or a young person not yet weighing 40 kg (and therefore still requiring ART adjustment based on weight), attending two visits in a year would indicate sub-optimal engagement because they should be seen in clinic more regularly. Our model is novel because for the first time it incorporates changes in HIV treatment guidance between paediatric and adult care and considers the clinical scenario of children and young people in the EIC measure. However, it does highlight the importance of linking YPLPHIV transitioning from paediatric to adult care in surveillance data sets (e.g. in the England setting from the Children’s HIV and AIDS Reporting System (CHARS) to the HIV and AIDS Reporting System (HARS)) to enable this work to be implemented moving forward.

Findings from this study suggest that most patients followed the scheduling requested by the clinician and were engaged in care. However, findings also demonstrated that participants did not stay on the same appointment schedule, with 37% of young people having at least one of their appointments predicted at 1 month, 40% at 2 or 3 months, 61% at 4 months and 24% at 6 months. This is an important public health message because it shows that it is hard to predict the care needs of these young people, as their needs may change over time. Our analysis only considered one year of follow-up, and if the analysis was over a longer period of time, or if indeed the whole of adolescence was considered, the pattern of appointment scheduling would likely vary even more. Furthermore, this finding supports the importance of a flexible measure of EIC that can pick up the nuances of clinical care in this age group.

In our study, we also observed that young people were most commonly predicted an appointment in 4 months’ time but that the median number of appointments was 4 visits per patient per annum (i.e. 3 monthly). This is most likely explained by the early attendance shown in the results. Reassuringly, when characteristics of participants who attended early were compared to those who did not, no differences were found. This early attendance therefore raises a question about the accuracy of the algorithm and whether doctors suggested visit scheduling less frequently than was actually undertaken in clinical practice. Alternatively it could suggest that doctors think that patients are able to be seen at longer intervals but that patients themselves wish to come earlier. Comparisons across appointment schedules should be viewed with caution because there is greater opportunity for participants to be early for a 6 month appointment than an appointment predicted in 3 or 4 months’ time. However, the reasons for early attendance could be further investigated as early attendance may have implications for costings and resources if people are being seen more often than needed based on their clinical status.

It is hard to directly compare our EIC findings to other studies due to considerable variability in how EIC is defined, in terms of the types of visits considered as either missed or attended (e.g. appointments with the doctor, nurse, psychologist or phlebotomist), and visit frequency (e.g. one visit in 6 months or one visit in a year [24]). In addition, many decisions underpinning EIC definitions are ultimately pragmatic and based on the clinical and appointment information available to researchers, as well as simplicity of approach to analysis. Nevertheless, it is important to try to contextualise findings. In our study, 87% of patient months of follow-up were defined as in care. European studies of YPLPHIV have reported engagement ranging from 80 to 98%, [23, 25, 26] and sub-Saharan African studies 83% to 98%, with studies commonly using a criterion of ≥ 1 visit in 6 or 12 months across varied age ranges [27–31]. Only one study has previously estimated EIC in YPLPHIV in the UK. Chappell et al.found that 98% of paediatric patients in the CHIPS study were engaged in care, defined as having a clinical visit, CD4 or viral load measurement, or ART change, within 2016 [23]. The median age was 14.4 years [IQR 11.2, 16.4], younger than in our study, and all children had to not be lost to follow-up in the previous three years to be included in the analysis.

The proportion of participants engaged in care in studies from the USA vary more than studies from Europe and sub-Saharan Africa, largely explained by the difference in EIC measures. Hussen et al. [22] found 56% of young people were engaged in care using a stricter multiple visit marker measure (≥ 2 visits with ≥ 3 months apart within 1 year), compared to Gray et al. [21] who found 99% of young people were engaged in care using a single visit measure (≥ 1 visit within 1 year).

The importance of a flexible EIC measure also has an increasing global relevance due to the roll out of differentiated service delivery across much of sub-Saharan Africa [32]. Differentiated service delivery is a person-centred HIV care approach that aims to increase patient choice around their interaction with the healthcare system, for example by providing options to attend community-based HIV care and/or reduce clinic visits by accessing multi-month dispensing of ART (compared to the more common model of monthly clinic visits for all) [32], However there remain limited data on the impact of differentiated service delivery on EIC and appropriate measurment will need to move away from simplistic measures to provide critical information for clinical care and policy globally. Currently very few countries have linked individual patient data across paediatric and adult health services that would allow this analysis to be carried out. Indeed in England, these data are not currently available outside of the AALPHI study, which is now out of date. We are currently working towards linking paediatric and adult surveillance datasets to allow future analyses across the lifespan. However, the process is lengthy due to complex governance issues.

A key question moving forward in the post-COVID landscape for all EIC measurements and studies is whether we are measuring what we need to measure. Many clinics now have a proportion of visits which are virtual, and this has implications for studies that do not collect data on dates of virtual visits, or indeed actual face to face visits, and instead rely on proxy markers such as viral load and CD4 counts to infer attendances (as in CHIPS). Clinicians, researchers, service providers and commissioners need to work together to ensure the collection and availability of the right data are possible to be able to measure this key HIV outcome.

### Limitations

There are a number of possible limitations to this analysis. Firstly, the participants included in this study are likely to be those young people who are more likely to be engaged in care as they were largely recruited into AALPHI from NHS clinics or voluntary sector organisations. The strict criteria used in these flowcharts and the requirement for each patient to fit into a terminal box somewhat limits the flexibility allowed within the context of individualised patient care. The criteria of time to next predicted appointment, as set out in the flowcharts, may not fully reflect national practice due to all clinicians involved in the development of the flowcharts being from large tertiary clinics; these clinicians may see stable patients less frequently. Our use of clinical markers to assemble proxy clinic appointments may have led to possible bias with potential for underestimation of attendances. Howarth et al. [14] highlight that one of the major weaknesses of their algorithm is that patients may visit HIV clinics for psychosocial issues which may not be captured in the clinical dataset, and this is relevant for our dataset too. A study could have been conducted to compare our dataset with clinic notes of actual attendances, but was not undertaken.

In addition to the methodological limitations, there are practical considerations and limitations of the flowcharts. Due to the number of criteria used in the classification of the follow-up of YPLPHIV, the flowcharts were complex and time consuming to set up and they require regular updates in accordance with changing guidelines, practice and treatment options. For example, these flowcharts focus on the treatment choice between the use of PIs and NNRTIs as third agents. Integrase inhibitors were not being routinely used in paediatric/adolescent care when the flowcharts were developed but now are recommended for first and second line therapy in all ages.

## Concluding remarks

Despite these limitations, these flowcharts have allowed detailed analysis of routinely collected healthcare data. If this model was regularly applied to surveillance data, results could be used to assess workload in clinic, predict costing, review resources allocation, benchmark between services and form a basis for future interventional studies. The adaption of the flowcharts provides a new approach to measuring EIC in YPLPHIV that is easy to understand and is reflective of changing patient needs over time. However, we acknowledge the complexity of these flowcharts would require ongoing investment which is a challenge in many settings.

### Supplementary Information

**Additional file 1. **Members of the Adolescents and Adults Living with Perinatal HIV (AALPHI) Steering Committee

## Data Availability

The datasets generated and/or analysed during the current study are not publicly available as they contain indirect personal identifiers, but are available from the corresponding author on reasonable request.

## Abbreviations

AALPHI: Adolescent and Adults Living with Perinatal HIV
ART: Antiretroviral therapy
BHIVA: British HIV Association
CDC: Centers for Disease Control and Prevention
CHIPS: Collaborative HIV Paediatric Study
EIC: Engagement in care
HIV: Human Immunodeficiency Virus
NNRTI: Non-nucleoside reverse transcriptase inhibitors
PI: Protease inhibitor
VL: Viral Load
YP: Young People
YPLPHIV: Young people living with perinatal HIV
