# Supplementary material for: An adapted algorithm for patient engagement in care for young people living with perinatal HIV in England
Source: BMC Health Serv Res. 2023 Oct 18;23:1114. doi: 10.1186/s12913-023-10122-5 (PMC10583428; doi:10.1186/s12913-023-10122-5)
Supplement: Supplementary file 1 — Additional file 1. Members of the Adolescents and Adults Living with Perinatal HIV (AALPHI) Steering Committee [file 12913_2023_10122_MOESM1_ESM.docx]

## Additional file 1: Members of the Adolescents and Adults Living with Perinatal HIV (AALPHI) Steering Committee

Project team: S. Brice, H. Castro, A. Judd, M. Le Prevost, A. Mudd, A. Nunn, K. Rowson, K. Sturgeon. Investigators: M. Conway, K. Doerholt, D. Dunn, C. Foster, D.M. Gibb, A. Judd (PI), S. Kinloch, N. Klein, H. Lyall, D. Melvin, K. Prime, T. Rhodes, C. Sabin,M. Sharland, C. Thorne, P. Tookey. MRCCTU Data Services: C. Diaz Montana, K. Fairbrother, M. Rauchen- berger, N. Tappenden, S. Townsend. Neurocognitive subgroup: A. Arenas-Pinto, H. Castro, C. Foster, A. Judd, M. Le Prevost, D. Melvin, A. Winston. Steering Committee chairs: D. Gibb, D. Mercey (2012–2015), C. Foster (2016–). Patient and public involvement: Children’s HIV Association Youth Committee NHS clinics (named alphabetically): LONDON: Chelsea and West- minster NHS Foundation Trust, F. Boag, P. Seery; Great Ormond Street Hospital NHS Foundation Trust, M. Clapson, V. Noveli; Guys and St Thomas’ NHS Foundation Trust, A. Callahgan, E. Menson; Imperial College Healthcare NHS Trust, C. Foster, A. Walley; King’s College Hospital NHS Foundation Trust, E. Cheserem, E. Hamlyn; Mortimer Market Centre, Central and North West London NHS Foundation Trust, R. Gilson, T. Peake; Newham University Hospital, S. Liebeschuetz, R. O’Connell; North Middlesex University Hospital NHS Trust, J. Daniels, A. Waters; Royal Free London NHS Foundation Trust, T. Fernan- dez, S. Kinloch de Loes; St George’s University Hospitals NHS Foundation Trust, S. Donaghy, K. Prime. REST OF ENGLAND: Alder Hey Children’s NHS Foundation Trust, S. Paulus, A. Riordan; Birmingham Heartlands, Heart of England NHS Foundation Trust J. Daglish, C. Robertson; Bristol Royal Infirmary, University Hospitals Bristol NHS Foundation Trust, J. Bernatonlene, L. Hutchinson, University Hospitals Bristol NHS Foundation Trust, M. Gompel, L. Jennings; Leeds Teaching Hospitals NHS Trust, M. Dowie, S. O’Riordan; University Hospitals of Leicester NHS Trust, W. Ausalut, S. Bandi; North Manchester General Hospital, Pennine Acute Hospitals NHS Trust, P. McMaster,K. Rowson; Royal Liverpool and Broadgreen University Hospitals NHS Trust, M. Chaponda, S Paulus. Voluntary services (named alphabetically): Blue Sky Trust, C. Dufton, B. Oliver; Body and Soul, A. Ash, J. Marsh; Faith in People, I. Clowes, M. Overton; Positively UK, M. Kiwanuka, A. Namiba; Positive Parenting & Children, N. Bengtsson, B. Chipalo. Lead contact for the AALPHI Steering Committee: Prof Ali Judd, a.judd@ucl.ac.uk.
